# Models for the Prediction of Receptor Tyrosine Kinase Inhibitory Activity of Substituted 3-Aminoindazole Analogues

**DOI:** 10.3797/scipharm.1102-08

**Published:** 2011-04-28

**Authors:** Monika Gupta, Harish Dureja, Anil Kumar Madan

**Affiliations:** 1 Faculty of Pharmaceutical Sciences, M. D. University, Rohtak-124001, India; 2 Faculty of Pharmaceutical Sciences, Pt. B. D. Sharma University of Health Sciences, Rohtak-124001, India

**Keywords:** Topological indices, Receptor tyrosine kinase inhibitors, 3-Aminoindazoles, Decision tree, Moving average analysis

## Abstract

The inhibition of tumor angiogenesis has become a compelling approach in the development of anticancer drugs. In the present study, topological models were developed through decision tree and moving average analysis using a data set comprising 42 analogues of 3-aminoindazoles. A total of 22 descriptors (distance based, adjacency based, pendenticity and distance-cum-adjacency based) were used. The values of all 22 topological indices for each analogue in the dataset were computed using an in-house computer program. A decision tree was constructed for the receptor tyrosine kinase KDR (kinase insert domain receptor) inhibitory activity to determine the importance of topological indices. The decision tree learned the information from the input data with an accuracy of 88%. Three independent topological models were also developed for prediction of receptor tyrosine kinase inhibitory (KDR) activity using moving average analysis. The models developed were also found to be sensitive towards the prediction of other receptor tyrosine kinases *i.e.* FLT3 (fms-like tyrosine kinase-3) and cKIT inhibitory activity. The accuracy of classification of single index based models using moving average analysis was found to be 88%. The performance of models was assessed by calculating precision, sensitivity, overall accuracy and Mathew’s correlation coefficient (MCC). The significance of the models was also assessed by intercorrelation analysis.

## Introduction

Cancer is a leading cause of death worldwide and accounted for 7.9 million deaths (around 13% of all deaths) in 2007. Moreover, the deaths resulting from cancer are expected to rise continuously with an estimated 12 million deaths in 2030 [1]. Cancer is thought to reflect a multistep process, resulting from an accumulation of inherited, acquired or both defects in genes involved in the positive or negative regulation of cell proliferation and survival. Activation or inactivation of just four or five different genes may be required for the development of clinically recognizable human cancer [2]. Despite advances in diagnosis and treatment, overall survival of patients still remains poor. Surgery, chemotherapy, radiotherapy, and endocrine therapy have been the standard options available for treatment of cancer patients. This has improved survival in several types of solid tumors, but treatment-related toxicity and emergence of drug resistance have been the major causes of morbidity and mortality [3]. Consequently, there is an urgent need to develop newer more effective therapies to improve patient outcomes. Blood is essential for solid tumors to manage nutritional supplies and waste removal to manage the tumor growth and metastasis. Angiogenesis is a process in which new blood vessels are formed from pre-existing vasculatures [4]. It has been reported that the angiogenesis is a rate limiting step in tumor development. Tumors that lack adequate vasculature become necrotic or apoptotic and don’t grow beyond a limited size [5, 6]. Inhibition of tumor angiogenesis has become a compelling approach in development of anticancer agents [7, 8]. Vascular endothelial growth factor (VEGF) is the primary endothelial cell specific angiogenic factor [9]. VEGF activity is mediated by three higher affinity receptors belonging to the class-V subfamily of receptor tyrosine kinases (RTKs). These are widely known for regulating angiogenesis, vasculogenesis, and lymphangiogenesis. The VEGFR family includes VEGFR-I/FLT-1 (fms-like tyrosine kinase-I), VEGFR-2/FLK-I (fetal liver kinase-I)/KDR (kinase inert domain containing receptor) and VEGFR-3/FLT-4 (fms-like tyrosine kinase-4). VEGFR-I is required for endothelial organization during vascular development while VEGFR-2 is required for formation of blood islands and also hematopoiesis [3, 5]. VEGFR-3 plays a significant role in VEGF-C and VEGF-D-mediated lymphangiogenesis. Over activation of KDR by VEGFs has been linked to progression of variety of human cancers. VEGF mediated KDR signaling induces a series of endothelial responses such as proliferation, migration and survival which ultimately leads to new vessel formation and maturation [10–13]. The immature tumor vasculature lacking a close association with pericytes appears to be most susceptible to inhibition of VEGFR signaling [14] and resistance to continued inhibition of VEGFR signalling has been reported [15]. Evidence increasingly points to a key role for PDGFR (platelet derived growth factor receptor tyrosine kinsases), located on the pericytes in the tumour stroma, in angiogenesis and vessel maturation and highlights its potential as a therapeutic target [16]. PDGFR is class III subfamily of RTKs including five receptors i.e. PDGFR-α, PDGFR-β, CSF-IR (colony stimulating factor 1 receptor), cKIT and FLT3 involved in cellular growth, differentiation, cytokine vascular regulation, gliomas and leukemia [3, 17]. FLT3 is shown to be commonly overexpressed in most B lineage acute lymphocytic leukemia (ALL), acute myeloid leukemias (AMLs) and chronic myeloid leukemias (CML) [18]*. Preclinical studies have already shown the benefits of combining VEGFR and PDGFR inhibition with respect to tumour response* [19]. Due to vital role that RTKs play in tumor angiogenesis, inhibition of these may prove to be an effective therapeutic intervention and potential inhibitors can be explored [2, 3].

The drug research and development is comprehensive, expensive, time-consuming and full of risk [20]. The traditional approach of drug discovery involves target identification, validation, lead search and optimization followed by clinical development phases [21]. The experimental search for better activities in drug discovery is commonly carried out in the laboratory by optimizing the structure–activity relationship (SAR) of the functional groups present in a leading structure in terms of their biological endpoint. However, an interesting alternative to this trial-error based procedure that constitutes an active field in complex biochemical phenomena are the analysis through Quantitative Structure–Activity/Property/Toxicity Relationships (QSAR/QSPR/QSTR) [22]. Quantitative structure-activity relationship (QSAR) represents an attempt to correlate structure descriptors of compounds with their biological activity [23, 24]. An important aspect of this method is the use of good structural descriptors that represent the molecular features responsible for the relevant biological activity [25]. The chemical graph theory is largely applied to the quantitative characterization of molecular structures for predicting physicochemical, pharmacological and toxicological properties using graph theoretical invariants [26, 27].The graph theoretical invariants have been termed as *topological indices* [28, 29]. The computation of TI is very swift and the TIs have the advantage of being true structural Invariants, which means that their values are independent of molecular conformations [25]. In last few decades, the *topological indices* have emerged as powerful tools for predicting biological activity of molecules, and lead identification forming an integral part of new molecular research [30–33]. In the present study, relationship of topological descriptors with KDR inhibitory activities of 3-aminoindazoles has been investigated using decision tree and moving average analysis. The proposed models were also evaluated for the prediction of FLT3 and cKIT inhibitory activities.

## Methodology

### Dataset

**Fig. 1.**
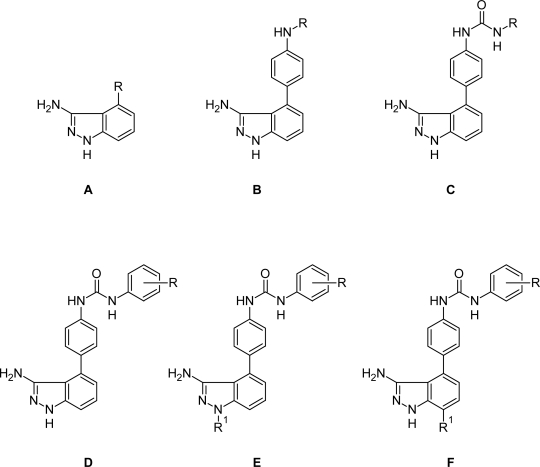
Basic structures of 3-aminoindazole analogues [34]

A dataset comprising of 42 analogues of substituted 3-aminoindazoles [34] was selected for the present investigations. The basic structure for these analogues is depicted in Fig. 1. and various substituents are enlisted in Tab. 1.

Kinase enzymatic assays of al the 42 analogues were preformed by Dai *et al.* [34] utilizing the homogeneous time-resolved fluorescence (HTRF) protocol. Peptide substrate at 4 μM, 1 mM ATP, enzyme and inhibitors (3.2 nM to 50 μM) were incubated for 1 hour at ambient temperature in 50mM NaOH (pH 7.5), 10mM MgCl_2_, 2mM MnCl_2_, 2.5 mM DTT, 0.1 mM orthovandate and 0.01% bovine serum albumin. The reactions were stopped with 0.5 M EDTA and then 75 μL buffer containing detecting agents (streptividine-allphycocyanin and PT66 antibody europium cryptate) was added. The plates were read from 1to 4 hour for time-resolved fluorescence. The inhibition was calculated using control and background reading. Each IC_50_ determination was preformed with seven concentrations and each assay point was reportedly determined in duplicate [34].

Subsequently, based on the results of KDR enzymatic assay, the potent inhibitors were reportedly characterized by cellular assay using 3T3 – murine fibroblasts cells [34].

The *in vivo* activity of compounds with potent cellular activity was also reportedly carried out using an estradiol-induced mouse uterine edema (UE) model. The said assay served as a valuable tool for a rapid and preliminary evaluation of KDR inhibitor’s oral activity.

The dataset comprised of variable degree of activities. Compounds having reported IC_50_ values of ≤ 10 nM were considered to be active while those possessing IC_50_ values >10 nM were treated to be inactive for the purpose of present study.

### Topostructural and topochemical indices

Twelve *topostructural* and ten *topochemical indices* [30, 35–55] used for the present study are presented in Tab. 2. The distance based topological descriptor (*Wiener index*, *Balaban index*), adjacency based descriptors (Zagreb group parameter, M_1_ and M_2_, *molecular connectivity index*) and distance cum adjacency based topological descriptors (*eccentric adjacency index, augmented eccentric connectivity index, superadjacency index, eccentric connectivity index, connective eccentric index, superaugmented eccentric connectivity index-2*) and pendenticity based descriptor (*superpendentic index*) were calculated using an in-house computer program. The topochemical versions of topostructural descriptors were calculated from distance and adjacency matrices weighed by molecular mass with respect to that of carbon atom.

### Decision tree

Decision tree provides a useful solution for many problems of classification where large datasets are used and the information contained is complex. A decision tree consisting of nodes and branches represents a collection of rules with each terminal node corresponding to a specific decision rule [56]. Decision trees are constructed beginning with the roots of tree and proceeding down to its leaves. In terms of ability, decision trees are a rapid and effective method of classifying data set entries and can provide good decision support capabilities [57, 58]. Applications of classification-based decision tree methods predominate in science and medicine [59]. It has been applied to some bioinformatics and cheminformatics problems, such as characterizations of tumor [60, 61], prediction of drug response [62], classification of antagonist of receptors [63] and identification of DNA sections coding exons [64].

In present study, decision tree was grown to identify the importance of *topological indices*. In a decision tree, the molecules at each parent node are classified, based upon the index value, into two child nodes. The prediction for molecule reaching a given terminal node is obtained by majority vote of molecules reaching the same terminal node in the training set. In this study, R program (version **2.1.0**) along with the RPART library was used to grow decision tree. The active compounds were labeled as “**A**” (n = 9) and the inactive compounds were labeled “**B**” (n = 33). Each analogue was assigned a biological activity which was then compared with the reported KDR inhibitory activity [34].

### Moving average analysis

Moving average analysis of correctly predicted compounds is the basis of development of single topological index based model [45, 65]. For the selection and evaluation of range specific features, exclusive activity ranges were discovered from the frequency distribution of response level and subsequently identify the active range by analyzing the resulting data by maximization of the moving average with respect to active compounds (<35% inactive, 35–65 % transitional, > 65 % active) The KDR inhibitory activity assigned to each compound was compared with reported biological activity. The various ranges obtained were also studied for the cKIT and FLT3 inhibitory activities. The average IC_50_ (nm) for each range and activity was also calculated.

### Data analysis

The sensitivity and specificity values were calculated which represents the classification accuracies for the active and inactive compounds, respectively. The randomness of model was also predicted by calculating Mathew’s correlation coefficient (MCC). The MCC values ranging between −1 to +1 indicates the potential of model. MCC took both sensitivity and specificity into account and it is generally used as a balanced measure in dealing with data imbalance situation [66]. The intercorrelation between M_2_^C^ and W_c_, and *^A^ξ^c^*^_2_^ was also investigated. The degree of correlation was appraised by correlation coefficient ‘r’. Pairs of indices with r ≥ 0.97 were considered highly inter-correlated, those with 0.90 ≤ r ≤ 0.97 were appreciably correlated, those with 0.50 ≤ r ≤ 0.89 were weakly correlated and finally the pairs of indices with r < 0.50 were not intercorrelated [67].

## Results and Discussion

**Fig. 2.**
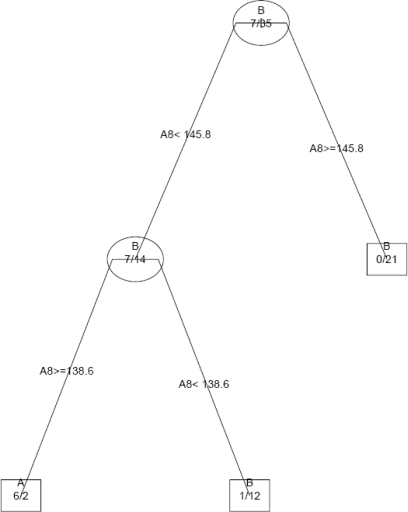
A decision tree for distinguishing active analogue (A) from inactive analogue (B); A8- Zagreb topochemical index, M_2_^C^

In the present study, decision tree was built from a set of 22 *topological indices*. The index at root node is most important and the importance of index decreases as the length of tree increases. The classification of 3-aminoindazoles analogues as inactive and active using a single tree, based on *Zagreb topochemical index* A8 is shown in Fig. 2.

The decision tree identified the *Zagreb topochemical index* A8 as the most important index. The decision tree classified the analogues with an accuracy of 88%. The precision and sensitivity of inactive analogues was of the order of 91.11% and 93.93%, whereas the precision and sensitivity of active analogues was of the order of 75% and 66.6% respectively (Tab. 3).

Three independent moving average analysis (MAA) based topological models were developed (Tab. 4.). The *topological index* A8, *Zagreb topochemical index* identified as most important index by decision tree was used to construct model for the prediction of KDR inhibitory activity. Two more indices *i.e. Wiener’s topochemical index*, A9 and *superaugmented eccentric connectivity index-2*, A20 were also used to develop the models for predicting KDR inhibitory activity. The methodology used in the present study aims at development of suitable models for providing lead molecules through exploitation of the active ranges in the proposed models based on topological indices. Proposed models are unique and differ widely from the conventional QSAR models. Both systems of modelling have their own advantages and limitations. In the instant case, the modelling system adopted has distinct advantage of identification of narrow active range, which may be erroneously skipped during routine regression analysis in conventional QSAR modelling [44]. Since the ultimate goal of modelling is to provide lead structures, therefore, these active ranges can play vital role in lead identification.

Retrofit analysis of the data reveals the following information with regards to different models used in this study. The biological activity was assigned to 42 analogues in both active and inactive ranges, out of which activity of 37 analogues was correctly predicted resulting in 88.09% accuracy with regard to KDR inhibition using model based on *Zagreb topochemical index* (A8).

**Fig. 3.**
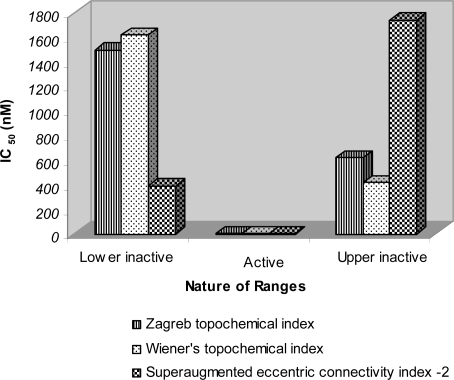
Average IC_50_ (nM) values of 3-aminoindazoles for KDR inhibitory activity in various ranges of topological models derived through moving average analysis

**Fig. 4.**
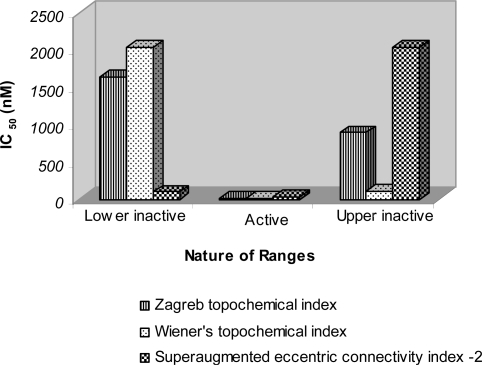
Average IC_50_ (nM) values of 3-aminoindazoles for FLT3 inhibitory activity in various ranges of topological models derived through moving average analysis

**Fig. 5.**
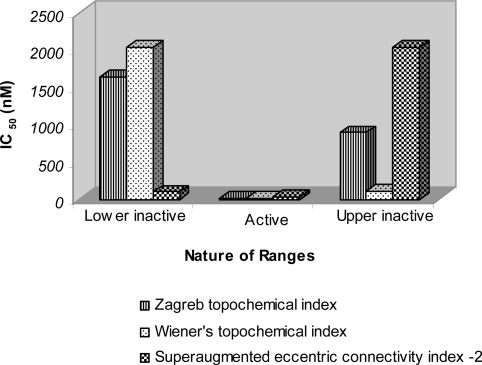
Average IC_50_ (nM) values of 3-aminoindazoles for cKIT inhibitory activity in various ranges of topological models derived through moving average analysis

31 out of 34 compounds (91%) in both the inactive ranges were predicted correctly. The average IC_50_ value of all the correctly predicted analogues in both the inactive ranges was 1494.5 nM and 615.421 nM respectively (Fig. 3). The average IC_50_ of correctly predicted analogues in the active range was found to be only 5 nM with regard to KDR inhibitory activity. Such a low average IC_50_ value signifies high potency of the active range. The said active range also exhibited significant FLT3 activity with average IC_50_ value of 19.86 nM (Fig. 4) and cKIT activity with average IC_50_ value of 19.714 nM (Fig. 5). The precision and sensitivity of inactive analogues was found to be 93.93% and 91.11%, whereas the precision and sensitivity of active analogues was of the order of 66.6% and 75% respectively. The 3-aminoindazoles analogues were correctly classified as active or inactive using *Wiener’s topochemical index* with an accuracy of 88%. The biological activity was assigned to 42 analogues in both active and inactive ranges, out of which 37 analogues were correctly predicted. 31 out of 34 compounds (91.1%) in both the inactive ranges were predicted correctly. The average IC_50_ value of all the correctly predicted analogues in both the inactive ranges was 1613.57 nM and 413.94 nM, respectively whereas the average IC_50_ of correctly predicted analogues in the active range was found to be only 5.33 nM (Fig. 3). Such a low average IC_50_ value signifies high potency of the active range.

The above active range also exhibited significant FLT3 activity with average IC_50_ value of 21.29 nM (Fig. 4) and cKIT activity with average IC_50_ value of 23.43 nM (Fig. 5).

The precision and sensitivity of inactive analogues was found to be 93.93% and 91.11%, whereas the precision and sensitivity of active analogues was of the order of 66.6% and 75% respectively.

*Superaugmented eccentric connectivity-2*, classified the 3-aminoindazoles analogues as active and inactive with an accuracy of 88%. The biological activity was assigned to 42 analogues in both active and inactive ranges, out of which 37 analogues were correctly predicted. 31 out of 34 compounds (91.1%) in both the inactive ranges were predicted correctly.

The average IC_50_ value of all the correctly predicted analogues in both the inactive ranges was 389.5 nM and 841.75 nM, respectively whereas the average IC_50_ of correctly predicted analogues in the active range was found to be only 6.33 nM (Fig. 3). Such a low average IC_50_ value signifies high potency of the active range. The said active range also exhibited significant FLT3 activity with average IC_50_ value of 25 nM (Fig. 4) and cKIT activity with average IC_50_ value of 31 nM (Fig. 5).

The overall accuracy of prediction was found to be 88%. The precision and sensitivity of inactive analogues was found to be 93.93% and 91.11%, whereas the precision and sensitivity of active analogues was of the order of 66.6% and 75% respectively.

The MCC value was found to be 0.633 (Tab. 3.) suggesting the randomness in the distribution of data. The result of intercorrelation analysis (Tab. 5) reveals that the *^A^ξ^c^*^_2_^ was not correlated with M_2_^C^ and W_c_ while the pair M_2_^C^ and W_c_ was found to be appreciably correlated

## Conclusion

Models based on all the three topological descriptors exhibited high degree of predictability with regard to KDR inhibitory activity using decision tree and moving average analysis. Moreover, the active ranges of the proposed models also exhibited significant FLT3 and cKIT inhibitory activities. A combination of VEGFR and PDGFR inhibitory activities will naturally be more beneficial for the treatment of tumors. High degree of predictability of the proposed models can provide valuable lead structures for the development of potent receptor tyrosine kinase inhibitors (RTKs).

## Figures and Tables

**Tab. 1.** Relationship between topological indices and KDR inhibitory activity

**Cpd. No.**	**Basic Structure**	**R**	**R_1_**	**M_2_^C^**	**W_c_**	***^A^_ξ_^c^*^_2_^**	**KDR inhibitory activity**			
							**Predicted using MAA models**			**Reported**
							**M_2_^C^**	**W_c_**	***^A^_ξ_^c^*^_2_^**	
1	A	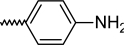	−	86.79	494.18	4.11	−	−	−	−
2	B	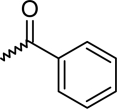	−	123.29	1674.22	2.02	−	−	−	−
3	B	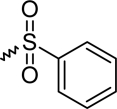	−	164.96	2093.78	2.15	−	−	−	−
4	B	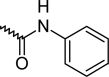	−	129.46	1949.52	1.76	−	−	−	−
5	C	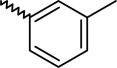	−	135.45	2172.95	1.81	−	−	−	+
6	C	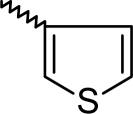	−	135.46	1750.1	1.98	−	−	−	−
7	C	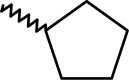	−	125.46	1729.09	1.98	−	−	−	−
8	C	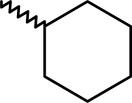	−	123.29	1674.22	2.02	−	−	−	−
9	D	2–F	−	136.20	2160.53	1.82	−	−	−	−
10	D	3–F	−	137.78	2180.53	1.81	−	−	−	−
11	D	4–F	−	137.79	2200.53	1.58	−	−	−	−
12	D	2–Me	−	134.45	2152.95	1.82	−	−	−	−
13	D	4–Me	−	135.46	2192.95	1.58	−	−	−	−
14	D	3–Et	−	139.46	2423.38	1.62	+	+	+	+
15	D	3–Cl	−	143.29	2198.40	1.81	+	−	−	+
16	D	3–Br	−	158.12	2246.62	1.81	−	−	−	−
17	D	3–CF_3_	−	152.70	2955.6	1.72	−	−	+	+
18	D	3–OH	−	136.79	2177.28	1.81	−	−	−	−
19	D	2–F–5–Me	−	141.20	2389.25	1.87	+	+	−	+
20	D	3–Me–4–F	−	144.37	2427.25	1.63	+	+	+	+
21	D	3–F–4–Me	−	144.37	2427.25	1.63	+	+	+	−
22	D	2–F–5–CF_3_	−	153.70	3191.65	1.78	−	−	−	−
23	E	3–Me	–Me	141.62	2383.54	1.73	+	+	+	+
24	E	3–Me	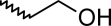	147.1	2635.78	1.67	−	−	+	−
25	E	3–Me	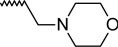	144.63	2391.41	1.73	+	+	+	+
26	E	2–F–5–Me	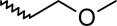	170.90	2460.04	1.73	−	+	+	+
27	F	3–Me	–Me	165.41	3909.63	1.43	−	−	−	−
28	F	3–Me	–OMe	173.57	4669.82	1.39	−	−	−	−
29	F	3–Me	–F	179.73	4664.31	1.41	−	−	−	−
30	F	3–Me	–Br	190.45	5587.48	1.28	−	−	−	−
31	F	3–Me	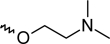	192.51	5554.98	1.36	−	−	−	−
32	F	3–Me	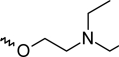	161.78	3572.18	1.42	−	−	−	−
33	F	3–Me	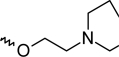	175.23	4126.54	1.5	−	−	−	−
34	F	3–Me	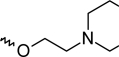	173.85	4123.64	1.5	−	−	−	−
35	F	3–Me	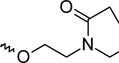	141.96	2409.05	1.54	+	+	−	−
36	F	3–Me	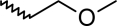	150.18	2924.31	1.42	−	−	−	−
37	F	3–Me	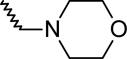	179.93	4595.26	1.28	−	−	−	−
38	F	3–Me	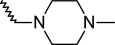	161.09	3501.97	1.54	−	−	−	−
39	F	3–Cl	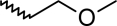	169.62	3602.53	1.42	−	−	−	−
40	F	2–F–5–Me	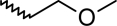	167.53	3873.60	1.45	−	−	−	−
41	F	2–F–5–Me	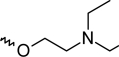	179.32	5030.37	1.41	−	−	−	−
42	F	2–F–5–Me	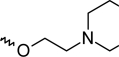	192.26	5466.49	1.37	−	−	−	−

+ Active analogue;

− Inactive analogue.

**Tab. 2.** Topostructural and topochemical indices

**Code**	**Index**	**Refe.**
A1	Molecular connectivity topochemical index	35,36
A2	Eccentric adjacency topochemical index	37
A3	Augmented eccentric connectivity topochemical index	38
A4	Superadjacency topochemical index	39
A5	Eccentric connectivity topochemical index	40
A6	Connective eccentricity topochemical index	41
A7	Zagreb topochemical index, M_1_^C^	42
A8	Zagreb topochemical index, M_2_^C^	42
A9	Wiener’s topochemical index	43
A10	Superaugmented eccentric connectivity topochemical index-2	44
A11	Molecular connectivity index	30
A12	Eccentric adjacency index	45
A13	Augmented eccentric connectivity index	46
A14	Superadjacency index	39
A15	Eccentric connectivity index	47
A16	Connective eccentricity index	48
A17	Zagreb index, M_1_	49,50
A18	Zagreb index, M_2_	49,50
A19	Wiener’s index	51,52
A20	Superaugmented eccentric connectivity index-2	53
A21	Balaban mean square distance index	54
A22	Superpendentic index	55

**Tab. 3.** Confusion matrix for KDR inhibitory activity using models based on decision tree

**Ranges**	**Number of cpds. predicted using decision tree**		**Precision (%)**	**Sensitivity (%)**	**MCC**
	**Active**	**Inactive**			
Active	6	3	75	66.6	0.63
Inactive	2	31	91.11	93.93	

**Tab. 4.** MAA based models for the prediction of receptors tyrosine inhibitory activity

**Model Index**	**Nature of range in proposed model**	**Index value**	**Number of analogues falling in the range**		**Overall accuracy of predict. (%)**	**KDR Aver. IC_50_ (nM)**	FLT3 Aver.* IC_50_ (nM)	cKIT Aver.* IC_50_ (nM)
			Total	Correct				
M_2_^C^	Lower inactive	<139.46	13	12		1379.77	84.54	1629.62
	Active	139.46–144.63	8	6	88.09	9.63	19.86	19.71
	Upper inactive	>144.63	21	19		557.76	189.31	893.14

W_c_	Lower inactive	<2383.54	16	14		1412.56	80.53	2029.19
	Active	2383.54–2460.04	8	6	88.09	9.87	21.29	23.43
	Upper inactive	>2460.04	18	17		391.5	207.86	108.73

*^A^ξ^c^*^_2_^	Lower inactive	<1.62	18	18		389.5	188.36	101.4
	Active	1.62–1.73	8	6	88.09	12.5	25	31
	Upper inactive	>1.73	16	13		1413.5	97	2037.25

* Average in a range is taken only for the reported IC_50_ values;

^#^Values in brackets are based upon correctly predicted analogues in the particular range.

**Tab. 5.** Intercorrelation matrix

	M_2_^C^	W_c_	*^A^ξ^c_2_^*
M_2_^C^	1	0.92	−0.73
W_c_		1	−0.68
*^A^ξ^c_2_^*			1
